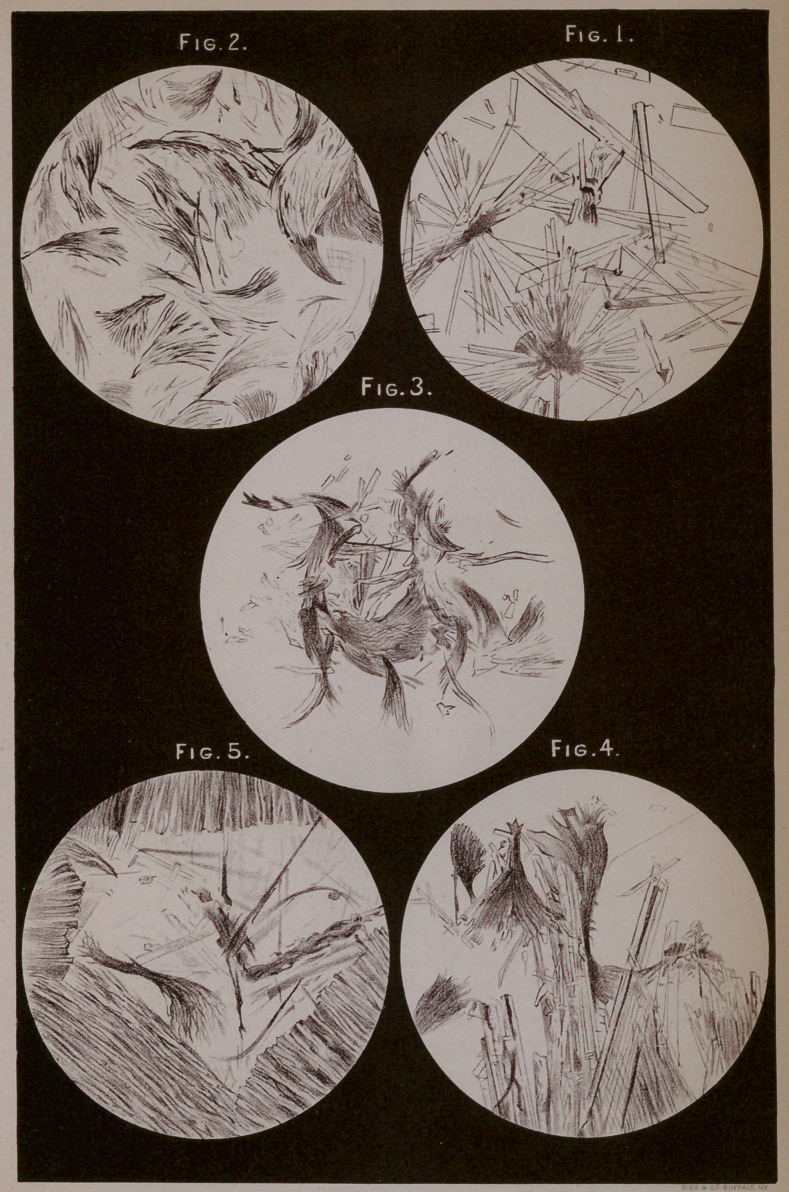# Butter, Fats: Their Adulteration and Methods of Examination with the Microscope

**Published:** 1886-06

**Authors:** Geo. E. Fell

**Affiliations:** Professor of Physiology and Microscopy, Medical Department, Niagara University


### *Butter, Fats: Their Adulteration and Methods of Examination with the Microscope.

By Geo. E. Fell, M. D., F. R. M. S.,

Professor of Physiology and Microscopy, Medical Department, Niagara University.

In a consideration of this subject, under the above heading, it may be appropriate to consider—

*Read before the Western Branch of the New York State Medical Association, Tuesday, May 11, 1886.

FIG. 2.

FIG. 1.

FIG. 3.

FIG. 5.

FIG. 4.

GIES & CO. BUFFALO, N.Y.

First.—The necessity for the use of the microscope in the discrimination of the various fats.

Second.—The differences in animal fats as revealed by the microscope.

Third.—The result of admixtures of different proportions of these fats, and its bearing upon the adulteration of butter.

Fourth.—The detection of adulteration and examination of butter by the methods of Dr. Thos. Taylor, Microscopist of the Agricultural Department at Washington, D. C.

From the results of an investigation into the adulterations of lard before the Chicago Board of Trade, in 1882 or 1883, it appears that the chemical methods in use at that time were not capable of detecting adulterations of lard with tallow. See Dr. Belfield's paper on "The Microscope in the Detection of Lard Adulterations," Chicago proceedings of the American Society of Microscopists. In this paper the statement is made that the chemical test is based upon the supposed difference in the relative proportions of stearine and olein in the two, that the beef-fat is supposed to contain more stearine than the hog-fat or lard, and that the chemical methods, based upon this assumed difference have, as yet, proved unsuccessful when put to a crucial test. In support of this the author of the paper gives an instance where "a chemist," who had sworn to his ability to detect the presence of tallow, when mixed with lard, failed completely. It is stated that "his analysis showed the utter worthlessness of the test," since he reported the discovery of tallow in two specimens which were known to be pure lard.

This article further states that some very high chemical authority, viz.: "Profs. Remson, of Baltimore; Doremus and Habirshow, of New York; Witthaus, of Buffalo, and Sharples, of Boston, all of whom testified upon this trial, stated that they knew no method whereby an adulteration of lard with tallow could be detected.

The application of the microscope to the detection of the differences in this case was based upon the fact that the dissolving of certain proportions of lard or tallow in Squibb's ether, with the subsequent evaporation of the ether, caused the deposition of peculiarly different crystals. These were indicative of the lard or tallow used; and admixtures of these substances showed the two forms of crystals with modifications of them, such as was satisfactorily determined to be sufficient to detect the presence of the lard or tallow, and to detect adulterations even with a percentage of adulteration as low as ten per cent. These statements were satisfactorily verified, so that now the question as to the value of the microscope in detecting such adulteration cannot be questioned. The plate reproduced from the proceedings of the American Society of Microscopists illustrates the differences between the crystals of lard and tallow, as found by the "ether method."

Pure lard, when treated by the ether method, throws down thin rhomboidal plates, whose obtuse angle measures about 105 degrees, see Fig. 1, while pure tallow, treated in precisely the same manner, gives, as in Figs. 2 and 3, curved, branching forms, very different and not to be at all compared with the lard crystals.

Various admixtures of lard and tallow exhibit, when treated by this method, the characteristics of lard and tallow as is seen in Figs. 4 and 5. This brings us to the second consideration in our paper, viz.:

The differences in the fats of various animals, as revealed by the microscope. It is found that some fat will readily crystallize when exposed to certain temperatures and conditions. Those in which the olein is in too great quantity, as lard, turkey fat, etc., require the removal of a portion of the oily product to enable crystallization to ensue at certain temperatures. The method employed by Dr. Taylor to obtain crystals of fats of different animals is to boil or render it at a suitable temperature, mix it with some suitable oil which will not interfere with its characteristic crystallization, or remove the superfluous olein by placing the fat, when melted, in an absorbent box or receptacle, which will absorb, in part, the oily products which interferes with crystallization. Outside of the general interest in the subject, much of the work done has been with reference to butter and its adulterations. On this account it is important to reduce the fats to the consistency of butter, when comparisons are to be made of the crystals. The details of this procedure will be given under the methods of detecting the adulterations of butter.

The differences in the fats of various animals, as shown by the variation in their crystals, will be based, in part, on a series of slides which I received through the kindness of Dr. Taylor within the last few weeks. The differences are indeed much greater than would be surmised, and a thorough consideration of this subject would be of very considerable scientific interest, and also may possibly result in information which may prove of practical value, even in fields foreign to the adulteration question—medico-legal for instance.

The fat of the musk-rat presents a large spicular crystal with spines radiating from different centers, and the majority of the crystals are made up of a great number of smaller ones, grouped together in an interesting manner.

Crystals of beef kidney fat are smaller, presenting radiating fan-shaped projections larger at the outer than at the central end, and not aggregated like the fat of the musk-rat.

The crystals of human fat are made up of much smaller forms of radiating spines, which present the larger end at the periphery of the crystal.

The crystals of dog fat has somewhat the appearance of the butter crystal, and the secondary crystallization begins with the formation of a rosette in the center of the mother crystal.

Lard is made up of small spicular crystals, radiating from a common center. Has no semblance to the butter or tallow crystal.

Mutton crystals closely similates lard, in that the crystals are spicular, radiating from a center, the spicules being smaller than those of lard on the slides examined.

The second stage of butter crystallization shows a coarser formation of the crystal than the first stage, with irregularly fan-shaped spines, and the margin of the crystal well defined.

The description of the crystals are incomplete, merely intended to give the general differences.

The result of admixtures of certain proportions of fats, with reference to its bearing on the adulteration of butter, will be indicated through the admixture of lard, beef-tallow, and butter as detailed herewith.

Equal parts of pure butter and lard were thoroughly commingled, allowed to cool, poured into a small box, and, after crystallization, examined. An occasional butter crystal was found, but by far most plentiful the characteristic crystals of lard. In fact, the lard appeared to predominate over the butter crystal, and was greatly in excess.

Equal parts of pure butter, beef-tallow and lard were melted together, allowed to cool, and, after crystallization, examined. The typical crystals of butter found in small numbers, the lard in profusion, and a few beef-tallow crystals were present.

Equal parts of pure butter and beef-tallow were melted together, put through a similar process; the crystals of tallow were found in profusion, but the characteristic features of the butter crystal was wanting.

Many other examinations of this nature were made, with similar results. The conclusion arrived at from the observations were certainly indicative of a marked variation from the characteristic appearance of the butter crystal.

As these fats (hog and beef fat) are those most frequently used in adulterating butter, the importance of such examinations is at once appreciated.

In looking over the N. I. Nathan process of making artificial butter—patent office reports—his claim is worded as follows:

"The within described process of manufacturing artificial butter, by uniting oleomargarine (in which tallow enters) with leaf lard, the latter having been previously cleaned, fused, strained, and subjected to a washing action in a solution of borax and nitric acid, then rewashed, and the united mass heated and subjected to the ordinary churning process, all substantially in the manner described," only indicates that lard and tallow are the common adulterants of butter.

As to the detection of adulterations in butter with the microscope, very little has been done, although reference is made to it in one of the later editions of "Hassall," a high English authority on food and its adulterations, but merely with the view of showing that such examinations at that time were not considered of special value; in fact, Hassall says that the "belief is entirely erroneous" that the crystals of butter and other fats are of value in detecting adulterations of butter.

With our present knowledge, we cannot agree with even so high authority as Hassall.

At the Cleveland meeting of the American Society of Microscopists, Dr. Thos. Taylor, of the Agricultural Department at Washington, read a paper entitled, "To Discriminate One Fat from Another by Means of the Microscope." His methods and results are concisely set forth in the following quotation:

"The experimenter should first procure a specimen of common lard. This is composed mostly of crystalline starry forms which represent the solid fat of the lard. Real lard is composed of these and the oil common to lard. In very hot weather, when the thermometer is up in the nineties, the crystals dissolve in the oil, and perfect crystals cannot then be obtained unless cooled slowly to about 70° F.

"Place a drop of sweet oil on a glass slide 3 x 1 inches, with the point of a needle. Place a small portion of the lard in the oil, and mix them together. Place a microscope glass disc over the lard and oil mixture, and press gently. If held up to the light white granules will be seen, if the temperature is not over 80° F.; these are fatty crystals. Under a low power of the microscope, it will be observed that these crystals have stellar forms with dark centers, and spines radiating from them.

"To procure normal crystals of beef kidney fat, render a piece of this fat in an iron pan, without water. Strain, and add sufficient sweet oil to bring the fat to the consistency of butter. Cool slowly for a period of from twelve to twenty-four hours. Mount in oil as directed in the case of lard. The crystals in this case present quite a different appearance from those seen in lard. View them by polarized light, with and without selenite plate. The beef crystals, to be seen to advantage, require a power of at least 500 diameters, being very small, although they appear very interesting objects with a power as low as 80.

"When it is desired to examine the crystals of butter, boil about an ounce of pure, newly-made butter in a test tube or iron spoon for a period of several seconds; allow it to cool as directed in the case of beef and lard; place a few grains of it on a slip of glass; pour over it a few drops of alcohol (or better alcohol nine parts, carbolic acid one part); separate the crystals with a pin, and view them with a pocket lens; they will appear like the eggs of insects. Place a second portion of the same butter on a glass slide 3 x 1 inches; combine it with a drop of sweet oil by means of a pin, reducing the butter to granules; cover with a thick disc of glass, and view first with plain transmitted light, when certain crystals will be seen. Second, by polarized light. In this case place the polarizer low down and turn the prism round until its face angle crosses the face angle of the analyzing prism above. Under these conditions a dark ground is produced, and the butter crystals, which are globular in form, are seen in bold relief. The butter globular crystals will now exhibit a well-defined black cross, representing that known as St. Andrews. If old butter or a poor, oily butter is used in this experiment, the secondary crystals of butter are generally shown. These crystals are of rosette form, much smaller than that of the globular, and exhibit no cross."

The simplicity of these methods, suggested by Dr. Taylor, and their importance, if found to be reliable, produced considerable interest regarding his work. At his request a committee was appointed by the Society to examine into his methods and report to the Society at an early date. The committee was made up as follows: Dr. H. J. Detmers, Professor of Veterinary Surgery, State University of Ohio, Columbus, chairman; George E. Fell, M. D., Professor of Physiology and Microscopy, Medical Department, Niagara University, Buffalo, N. Y.; Lester Curtis, M. D., Professor of Histology, Chicago Medical College, Chicago, Ill; C. M. Vorce, Esq., Cleveland, O.; and Mr. H. F. Atwood, of Rochester, N. Y., secretary of committee.

These gentlemen have all, it is believed, investigated the subject in various ways, doing a large amount of work, but no report has been yet made.

Mr. Atwood expresses himself to the effect that the methods are reliable and valuable, while from a letter Mr. Vorce sent me I glean his views more fully, and, as they are of general interest, I quote them in full.

Mr. Vorce expresses himself as follows:

"I have never yet seen a sample of oleomargerine or anything else that could, by any ordinary expert microscopist, be mistaken for butter, especially if the ether test be applied as a secondary test. Even by Dr. Taylor's method the difference between *pure* butter and any imitation is very marked. When pure butter is boiled and well drained, the residium, when mixed with olive oil, is seen to consist *entirely* of the so-called "typical crystals," showing, if dry-fed, a large, pale-yellow, smooth-edged crystal, showing, *in every crystal*, a revolving four-armed cross, the arms much narrower than the intervening segments, and radially widening to the edge. In grass-fed butter the crystals are smaller, darker, less regular, and not so smooth-edged, but in every other respect are the same. This is the result of my examination of about forty samples. In *all* the butter substitutes and imitations I have examined, there is a great deal of what might be called "refuse," such as granular matter, separate spicules of crystalline matter, oil globules, shreds, etc., in addition to any round or globular crystals which may be present. But I have never seen a crystal like that of butter in any oleomargerine, although I have in the *mixtures* of butter and lard; also in the butterine made under the patent of May, 1881, that under which cotton-seed oil, neat's-foot oil, etc., is used."

My own work in this connection has resulted in convincing me that much value may be placed upon Dr. Taylor's work. While in some cases the black cross does not appear upon pure butter, the absence of the crystals foreign to butter, and the results of examination with polarized light, are all additional aids in investigations of this nature. These views have been the result of examination of a large number of specimens of butter, lard, tallow, of known purity, and under conditions similating those in adulterations.

Many experiments have been made by observers throughout the country, through the publications of Dr. Taylor's paper; some of these are calculated to weaken his statements and lesson their value in a marked degree.

Professor H. A. Weber, of the Ohio Agricultural Experiment Station, at Columbus, has made a series of experiments in which he claims to have proved the worthlessness of the experiments of Dr. Taylor. He mixed lard, tallow, butter and oleo-oil together and separately, with sodium chloride in different proportions, and claims to have obtained the characteristic crystals of butter under such conditions as to stamp Dr. Taylor's work as valueless. He concludes his paper as follows: "Having thus described the production and nature of Dr. Taylor's "butter crystals," and having shown in the experiments given above that they are not peculiar to butter, but that mixtures of tallow, fat and lard, under the conditions which obtain in butter-making cannot by this means be distinguished from pure butter, it follows that so much of Dr. Taylor's microscopic investigations as pertain to the formation of characteristic crystals of these various fats, is of no practical value in the examination of commercial butter for adulteration."

Prof. Weber reported some fifteen experiments. Of these, Dr. Taylor answers and reviews the fourth as follows: "According to his report he made fifteen experiments in all. Of these only the first three have any relation to my methods. *The remaining twelve are based on his uniform plan of boiling all of his combinations of butterine at a high temperature.* Let us review experiment No. 4. He says: "A mixture consisting of ninety per cent. butter, and ten per cent. "oleo-oil" (beef-fat oil) was boiled and cooled as before. The mounted slides could not be distinguished from the pure butter slides." (He means that they showed only the butter cross). Why should he *boil* this oleomargarine when by simply mounting a portion and viewing it with polarized light and selenite plate the prismatic colors, which show the presence of foreign fats—the object to be attained—are at once observed? I have just examined a composition of butter and "oleo-oil" (beef-fat oil), in the proportions given above. The butter was melted at a temperature of 104 *degrees Fahrenheit*, and combined with the beef-fat oil. *The mounted slide shows no butter* crystals while it did exhibit *those of beef-fat* (oleo-oil). In this case I imitated the methods of those engaged in the production of butterine and oleomargarine by only *melting* the *butter* and *fats* at a *very low temperature.* Prof. Weber *erred* in *boiling* his oleomargarine, and this holds good with all his experiments from No. 4 to No. 15 inclusive. *His last twelve experiments must be eliminated from* the consideration of this subject, because they have no relation to *my methods of discriminating between butter and oleomargarine;* nor do the characteristics of the butter crystal, of necessity, have direct relation to the detection of oleomargarine. It is only when the *differentiation* of the respective crystals of the fats of the various plants and animals is considered, that the cross of butter becomes an important factor."

"In an experiment made as late as the 20th of this month (March), in the presence of Professor Wiley, Chief Chemist of the Department of Agriculture, and two of his assistants, Mr. Richardson and Mr. Richards, I demonstrated that a butterine, made at my request in the laboratory, which contained but *one per cent. of lard to ninety-nine per cent. of pure butter* exhibited, when examined by *polarized light* and *green selenite*, well-defined *crystals of lard*, of a *rich golden color* on a *green ground*, the green representing the butter. In this way the *adulteration was at once detected.*"

"The experiments of Professor Weber are calculated to introduce confusion and difficulty into a matter in itself comparatively clear and easy. In short, the object on which his efforts have been concentrated, like that so sedulously kept in view by the British Circumlocution Office, as portrayed by Dickens, appears to have been 'how *not* to do it.'"

"It must be borne in mind that the manufacturers of counterfeit butter are obliged to limit their treatment of the substances they use to such manipulations as are consistent with the salability of the product."

In addition to this, Mr. Vorce, of Cleveland, writes that he has made all the experiments of Professor Weber with some oleo-oil obtained from the latter, and finds that the crystals so prepared are not similar to those of butter, or that the difference is sufficient to enable a careful observer to distinguish between them. I have been unable to examine this phase of the question, but propose to do so before the next meeting of the American Society of Microscopists, which meets at Chautauqua next August, when the subject will undoubtedly be fully considered in open session.

In closing, let me voice my objection to the unjust legislation which Congress appears to be ready to take in *virtually* prohibiting the manufacture of butterine and oleomargarine. These products, in my opinion, when properly manufactured, are as wholesome articles of food as butter, and will replace, in many cases, if sold for what *they are*, the lard now used on the tables of the poor. Let the power of the law be invoked to control their manufacture and sale, but not tax to prohibition articles of inestimable food value.

## Figures and Tables

**Fig.1. Fig.2. Fig.3. Fig.4. Fig.5. f1:**